# Neurobehavioral Effects of Levetiracetam in Brain Tumor Related Epilepsy

**DOI:** 10.3389/fneur.2013.00099

**Published:** 2013-07-22

**Authors:** Abigail Bernett, Richard Phenis, Ekokobe Fonkem, Jose Aceves, Batool Kirmani, Daniel Cruz-Laureano

**Affiliations:** ^1^Division of Neuropsychology, Scott & White Memorial Medical Center, Temple, TX, USA; ^2^The Brain Tumor Center, Scott & White Memorial Medical Center, Temple, TX, USA; ^3^Texas A&M Health Science Center, Temple, TX, USA; ^4^Pediatric Neurology, Scott & White Neuroscience Institute, Temple, TX, USA; ^5^Epilepsy Center, Scott & White Neuroscience Institute, Temple, TX, USA

**Keywords:** levetiracetam, brain tumors, seizures, neurobehavioral

## Abstract

The neurobehavioral profile of anti-epileptic drugs (AEDs) has been a recurrent research topic in the scientific literature. As pharmacological treatments for epilepsy continue to evolve, there is a general consensus that newer AEDs have less detrimental side effects in comparison to their older counterparts. Among newer AEDs and epilepsy patients, potential risk for neurobehavioral changes has been reported with levetiracetam (LEV). Conversely, limited data exists regarding the manifestation of this symptomatology in a subgroup of epilepsy patients with brain tumors. The current paper reviews the literature regarding the neurobehavioral profile of LEV in brain tumor related epilepsy and suggestions for future research will be discussed.

## Introduction

The neurobehavioral profile of anti-epileptic drugs (AEDs) has been a recurrent research topic in the scientific literature. As pharmacological treatments for epilepsy evolve, there is a general consensus that newer AEDs have less cognitive side effects in comparison to their older counterparts. Levetiracetam (LEV) has garnered interest as a treatment for patients with brain tumor related epilepsy. Among patients with epilepsy, potential risk for neurobehavioral changes has been reported with LEV. Conversely, limited data exists regarding the manifestation of this symptomatology in epilepsy patients with brain tumors. The current paper reviews the literature regarding the neurobehavioral profile of LEV in brain tumor related epilepsy and suggestions for future research will be discussed.

## Anti-Epileptic Drugs

The adverse effects of AEDs have been fairly well documented in the scientific literature and can encompass both physical and neurobehavioral phenomena that can vary in their intensity and their impact on patients’ quality of life (1). A meta-analysis conducted by Zaccara et al. (2) revealed somnolence, dizziness, ataxia, diplopia, and fatigue to be the most common adverse physical symptoms of AEDs. Studies have shown that AEDs can negatively impact multiple cognitive domains (3). Further, depending on the choice of medication, AEDs may positively or negatively affect mood. Adverse psychiatric effects often include behavioral changes, depression, and psychosis (4). A benefit of the newer generation AEDs, such LEV, is that they tend to have less severe side effects and a decreased potential to negatively interact with other medications (5). As a result, there is a proclivity to consider newer generation AEDs as the first line of treatment for diverse epileptic syndromes, including brain tumor related epilepsy.

## Brain Tumor Related Epilepsy

Seizures are often the initial presenting symptom of intracranial tumors (6, 7). In a meta-analysis of prospective randomized clinical trials involving newly diagnosed brain tumors, Glantz et al. (8) found a 26% incidence of seizures occurring prior to diagnosis and the rates can be even higher depending upon tumor grade (9, 10). The incidence of brain tumor related epilepsy varies based on the type of tumor involved and there is an inverse correlation between tumor grade and likelihood of seizures (7, 11). The mechanisms behind brain tumor related epilepsy are somewhat unclear, although there are a number of proposed etiologic mechanisms including tumor location, histopathological features, the neurochemical profile of the tumor, and changes in neurotransmitter and receptor expression, among others (12).

Brain tumor related epilepsy is characterized by its intractability to anti-epileptic drugs and prior findings suggest that prophylactic AED use is not effective (10). The AAN Quality Standards Subcommittee meta-analysis regarding prophylactic use of AEDs determined that it does not provide substantial benefit and adverse effects are common (8). Another concern with use of AEDs in brain tumor related epilepsy is drug–drug interactions between AEDs and chemotherapeutic agents. Many older AEDs (e.g., phenobarbital, phenytoin, carbamazepine) are hepatic enzyme inducers, which is problematic because several chemotherapeutic agents are metabolized by hepatic chromosome P450 enzymes. AEDs that induce hepatic enzymes increase the metabolism of the chemotherapeutic agent, requiring the chemotherapy dose to be increased in order to maintain required blood levels (11, 13). Enzyme inducing agents can also interact with steroids that are commonly used to treat edema in patients with brain tumors (14). As a result of these drug interactions, newer generation AEDs that do not have hepatic enzyme inducing properties, such as LEV, have been identified as a pharmacological alternative for treating brain tumor related epilepsy (15).

## Levetiracetam

Levetiracetam’s mechanism of action is unclear, though some research has supported the idea that it is involved in the expression of synaptic vesicle protein 2A (SV2A) (12) and animal models have shown that SV2A is the binding site for LEV (16). Patsalos (17) noted that the pharmacokinetic profile of LEV meets nearly all the criteria that make an AED “ideal” because of its rapid absorption, limited metabolism, and low risk of drug interactions. It does not inhibit or induce hepatic enzymes like many of the older generation AEDs, which decreases the likelihood of interaction with other drugs. LEV has not been found to induce or inhibit metabolism in other AEDs or non-AEDs, such as chemotherapeutic agents (18). Bobustuc et al. (19) found that LEV sensitized glioblastoma to the chemotherapeutic agent temozolomide in a sample of four newly diagnosed patients, which provides evidence for additional properties of LEV that make it a good alternative for use in brain tumor related epilepsy.

In terms of efficacy, multiple studies have found LEV to reduce seizure frequency by 50% or more in the majority of brain tumor patients when used as monotherapy and/or as adjunctive treatment (6, 20–22). One study compared a group of 105 patients receiving LEV monotherapy with 210 patients receiving phenytoin monotherapy and found both drugs to be effective for early and late post-operative seizures (23). Lim et al. (24) looked at the safety and efficacy of switching patients from phenytoin to LEV following craniotomy in a sample of 29 patients and the two drugs had similar numbers of seizure free patients at 6 months. In addition to its efficacy regarding seizure control, other factors, such as its neurobehavioral profile, need to be considered.

## Neurobehavioral Profile of Levetiracetam

The literature regarding LEV’s neurobehavioral profile in epilepsy has been extensively reviewed (3, 17, 25, 26) and is particularly notable for behavioral changes (27) and increased aggression (28, 29). In particular, patients with premorbid depression or behavioral problems are at greater risk for developing increased aggression (4, 29). In addition, suicidality in some patients prescribed LEV has been reported (30).

Levetiracetam is not generally associated with cognitive side effects (3, 25). Some of the research on LEV and brain tumor related epilepsy has focused primarily on its efficacy related to seizure control and limited information regarding other adverse side effects is reported (22, 31). For the current paper, 14 studies were reviewed that reported on specific physical, behavioral, and cognitive side effects among patients with brain tumor related epilepsy treated with LEV.

Lynam et al. (6) conducted a retrospective analysis of 147 patients with newly diagnosed primary and metastatic brain tumors. Forty one of the patients were on LEV and the most commonly reported side effects included depression, fatigue, and irritability. A limited number of cases had side effects severe enough to discontinue the medication, though the exact number of cases went unreported (6). Newton et al. (32) conducted a retrospective chart review of 41 patients with brain tumors (34 primary brain tumors, 6 metastatic brain tumors) who received LEV as an add on or monotherapy and were followed for 4 weeks. In terms of side effects, 58.5% of the sample experiences side effects, and those on 2,000 mg or more were more likely to experience side effects. Somnolence was the most frequently reported side effect and other symptoms included dizziness, headache, and paresthesias. One patient discontinued LEV after developing panic attacks. This research group conducted a second chart review with a sample of patients with metastatic brain tumors (33) and reviewed the charts of 13 patients receiving LEV as monotherapy (46%) or add-on therapy (54%). At 4 weeks follow-up, 46 percent of the sample had adverse side effects including somnolence, headache, blurry vision, and nausea and vomiting.

Rosati et al. (34) conducted a prospective study with 176 newly diagnosed glioma patients. All patients diagnosed with epilepsy (47% of the sample) were initially treated with LEV. Two patients discontinued LEV during the study because of intolerable diarrhea and visual hallucinations with psychotic thoughts. de Groot et al. (20) also looked at patients with glioma and one patient from their sample of 35 discontinued LEV before follow-up data were gathered due to side effects including leukopenia. Wagner et al. (35) conducted a feasibility study looking at LEV in primary brain tumor patients. Their sample included 26 patients. Thirty-five percent of the sample reported side effects which most frequently consisted of fatigue, somnolence, and dizziness. One patient discontinued LEV after developing psychosis. Maschio et al. (21) also conducted a prospective study exploring the effectiveness of three newer AEDs (LEV, oxcarbazepine, topiramate) in a group of 30 patients with brain metastases. Six of the patients were taking LEV. Only three patients from the sample developed mild side effects. Two of the three were taking LEV and developed mild and reversible restlessness and rash.

Two comparative studies were found that compared LEV to phenytoin (22, 24). One study compared a group of 105 patients receiving LEV monotherapy with 210 patients receiving phenytoin monotherapy and found the patients prescribed LEV had fewer side effects. One patient discontinued LEV due to developing visual hallucinations, and 38 patients discontinued phenytoin due to various side effects (22). Another study that compared LEV to phenytoin included 29 patients post-craniotomy who were randomized into either a phenytoin group or a LEV group. At 3 months follow-up, patients using LEV were reporting fewer side effects in many areas, though they endorsed more difficulty sleeping (33% of LEV subjects) and emotional instability (13%) as compared to the patients in the phenytoin group [(24), p. 353]. One patient reported increased hostility toward others after beginning LEV, though this was not severe enough to discontinue the medication. Zachenhofer et al. (36) retrospectively studied 78 brain tumor patients receiving LEV following surgery. Side effects were reported in 6.4% of the sample, with three patients reporting progressive somnolence and two patients developing reactive psychosis.

Two studies were reviewed that included screens of cognitive functioning in their assessment of side effects. Usery et al. (37) completed a prospective open-label study looking at the safety and tolerability of intravenous and oral LEV following neurosurgery. The study was hindered by a small sample and only included 17 patients. The patients were followed for 1 month after discharge and six adverse events were reported including three episodes of somnolence, and one episode each of nausea/vomiting, headache, and insomnia. The researchers also used the Telephone Interview for Cognitive Status (TICS) (38) to evaluate weekly for any cognitive changes secondary to LEV. Two patients were deemed cognitively impaired due to their scores on this measure. It should be noted that the TICS is normed for individuals aged 60–98 years, and the current sample’s mean age was 56 with a range from 27 to 77. This would suggest that some of the participants were younger than the normative sample of the TICS, and thus their scores may be inaccurate.

Dinapoli et al. (39) conducted a case series to evaluate LEV as a monotherapy for brain tumor related epilepsy in a sample of 18 patients that they followed for 6 months. The Mini Mental Status Examination (MMSE) (40) was used as a measure of global cognitive function and the Karnofsky Performance Scale (KPS) (41) and the Bartel Index (BI) (42) were used to evaluate overall functioning. Sixty one percent of the sample had side effects at the beginning of the trial including rash, somnolence, periarthritis, weight loss, and liver toxicity. At 6 months 22.2% had mild side effects including somnolence and restlessness. At 6 months scores on all three measures were significantly worse than at baseline, though the MMSE mean score was still in the normal range (KPS 85.83, BI 87.78, and MMSE 26.78). This research group has also published two other studies, one of which followed 29 patients for 12 months (43). In terms of side effects, three patients reported restlessness, and one discontinued LEV due to the severity of the restlessness. Two patients reported somnolence. An earlier study by these researchers (44) looked at 19 patients with supratentorial gliomas and found the majority of patients had improved seizure frequency and no adverse side effects were reported.

## Conclusion

The existing research regarding LEV and brain tumor related epilepsy appears to provide support for the drug’s efficacy in reducing seizures. However, its neurobehavioral profile in this population remains somewhat unclear and the existing research has primarily focused on the drug’s anti-epileptic properties and tolerability. Given that LEV has a well-established neurobehavioral profile in the general epilepsy population, namely associated behavior change and increased aggression, it is important to examine whether a similar profile appears in patients with co-morbid neurological conditions (e.g., tumors). Several studies were reviewed that reported on side effects seen in brain tumor samples and the results varied widely, ranging from no side effects (44) to 58.5% of a sample reporting side effects (32). In terms of neurobehavioral changes, irritability (6), emotional instability (24), and restlessness (39) were seen in some patients. Interestingly, only once study mentioned a participant developing increased hostility toward others, though it was not severe enough to discontinue LEV (24). Multiple studies had patients who discontinued LEV due to significant psychiatric symptoms including panic attacks and psychotic symptoms (23, 32, 34–36).

The existing research has a number of limitations that make it difficult to draw conclusions across studies. Many of the studies were retrospective chart reviews and sample sizes tended to be quite small. Only one study was found that was prospective and randomized (37) and the study was closed early after only obtaining 17 participants.

Side effect data was typically gathered by self-report and focused primarily on health status (e.g., the Adverse Events Profile). Only two studies were found that included a brief cognitive screening instrument (37, 39) and no research studies included formal neuropsychological testing. In general, the focus of the existing research tended to be on seizure reduction while other areas were not fully explored.

Additional research is necessary to identify the neurobehavioral profile of LEV in brain tumor related epilepsy, as general conclusions cannot be drawn from the existing research. LEV does appear to have some support for its efficacy in terms of reducing seizure frequency and may have fewer side effects in comparison to other AEDs (23). However, the side effects reported across different studies varied widely, and it is unclear if there are particular patient characteristics (e.g., tumor location) that might interact with the drug of choice. Additional research that more fully explores the neurobehavioral effects of LEV in patients with brain tumors would be useful in order to improve patient care and allow providers to make a more fully informed decision when choosing a treatment option.

## Conflict of Interest Statement

The authors declare that the research was conducted in the absence of any commercial or financial relationships that could be construed as a potential conflict of interest.
